# Supplementary material for: Microwave-Assisted Synthesis of Mono- and Disubstituted 4-Hydroxyacetophenone Derivatives via Mannich Reaction: Synthesis, XRD and HS-Analysis
Source: Molecules. 2019 Feb 7;24(3):590. doi: 10.3390/molecules24030590 (PMC6384783; doi:10.3390/molecules24030590)
Supplement: Supplementary file 1 [file molecules-24-00590-s001.pdf]

Microwave-Assisted Synthesis of Mono- and Disubstituted 4-Hydroxyacetophenone Derivatives via Mannich Reaction: synthesis, XRD and HS-analysis

Ghadah Aljohani, Musa A. Said*, Dieter Lentz, Norazah Basar, Arwa Albar, Shaya Y. Alraqa, Adeeb Al-Sheikh Ali*

*Correspondence: musa_said04@yahoo.co.uk, AdeebAli@Dal.Ca

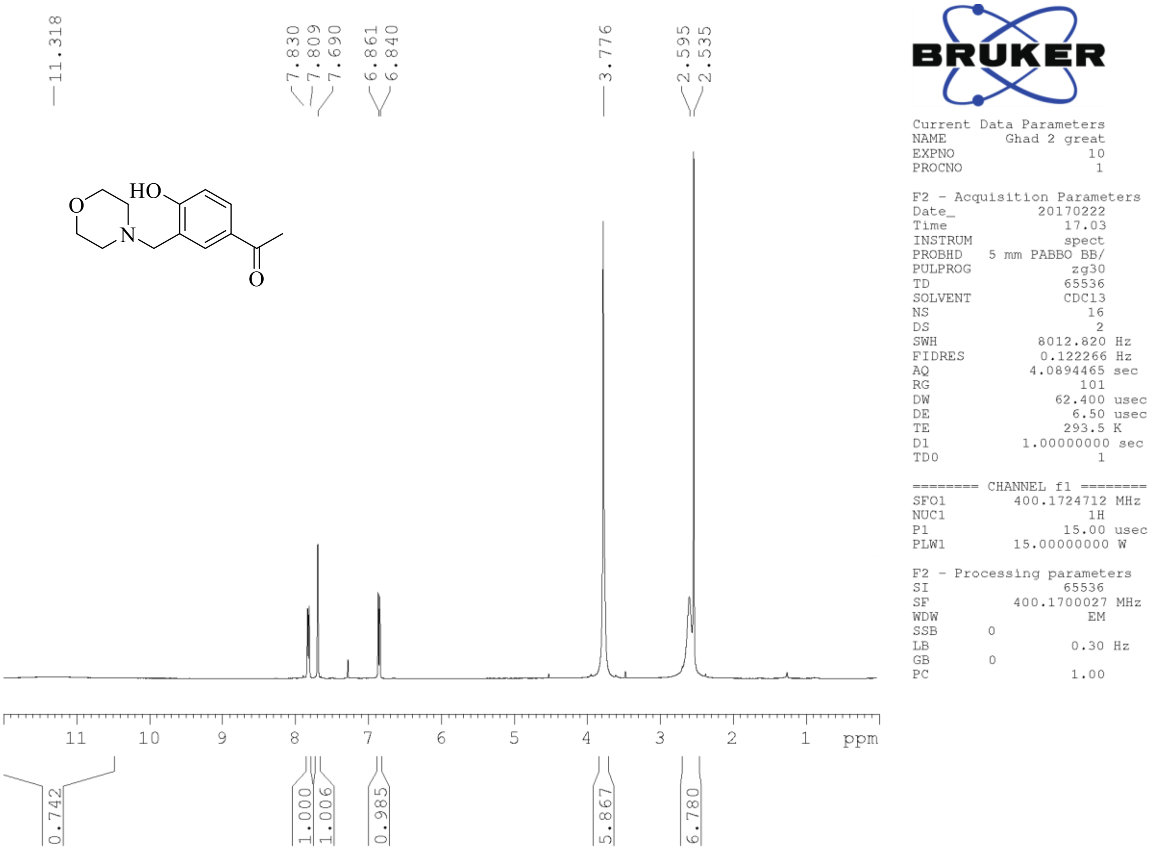

Figure S1. ^{1}HNMR spectrum of compound 2a in $CDCl_3$.

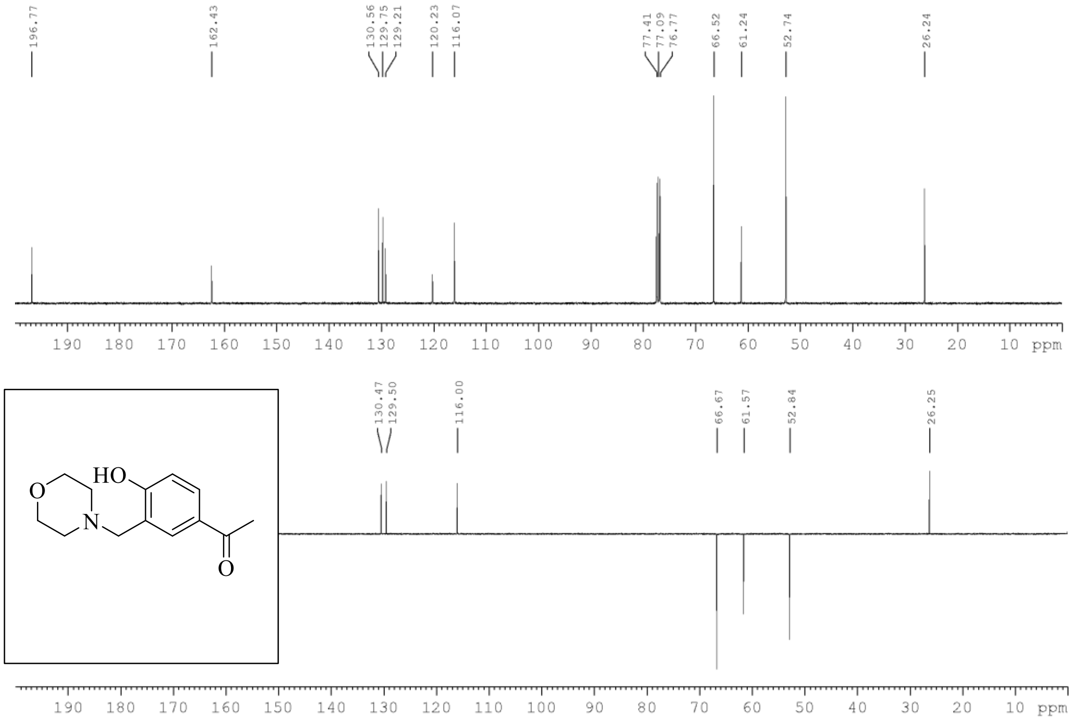

Figure S2. ^{13}C NMR &Dept 135 spectrum of compound 2a in $CDCl_3$.

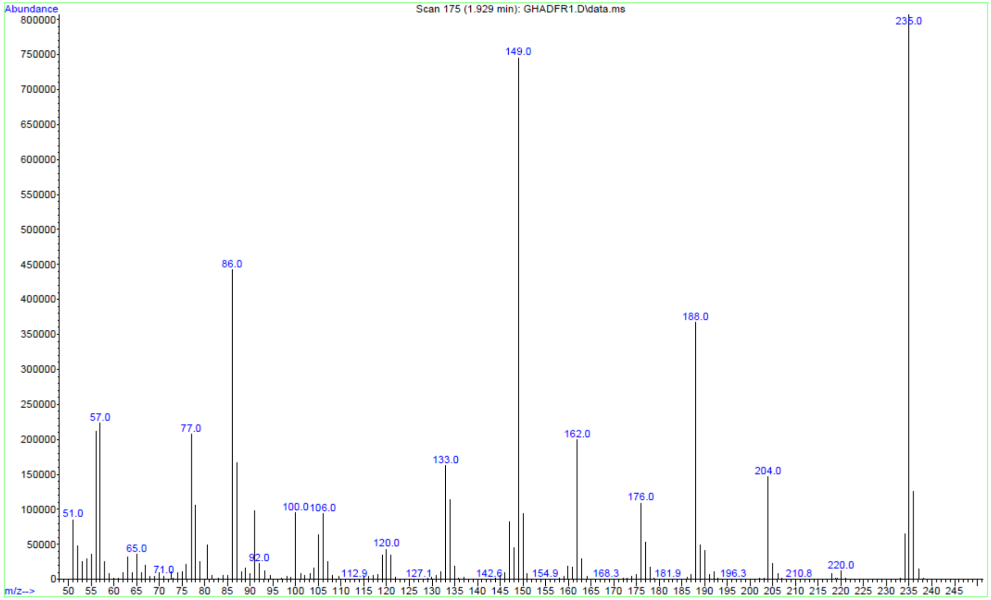

Figure S3. MS spectrum of compound 2a

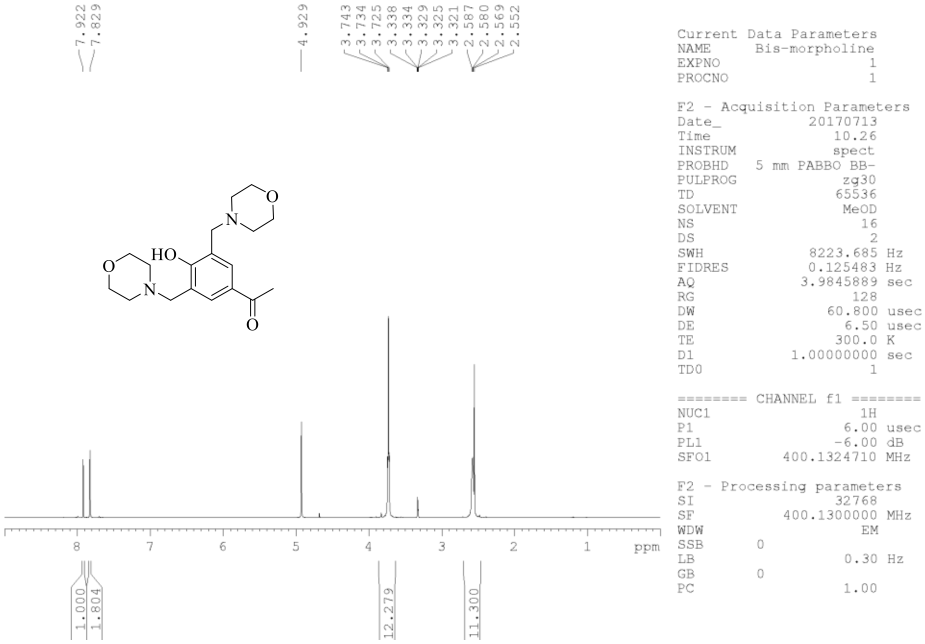

Figure S4. ^{1}HNMR spectrum of compound 2b in Methanol-d_4

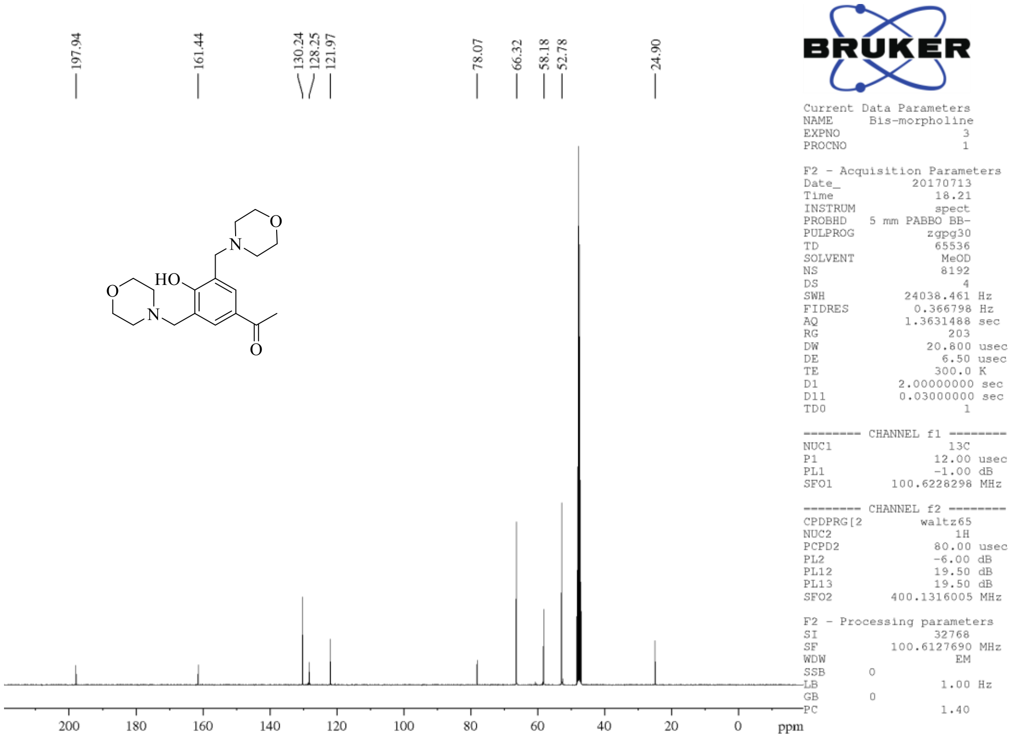

Figure S5. ^{13}C NMR spectrum of compound 2b in Methanol-d_4

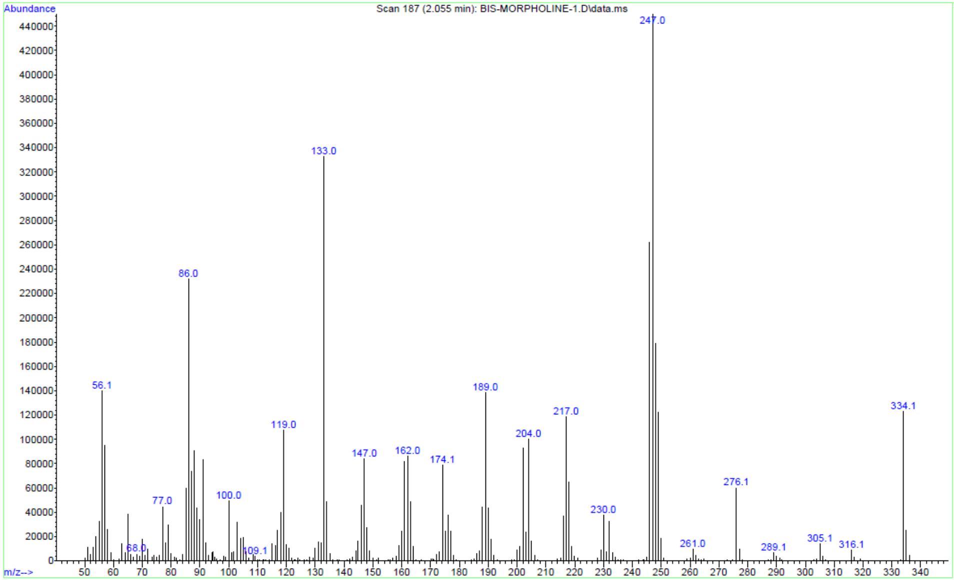

Figure S6. MS spectrum of compound 2b

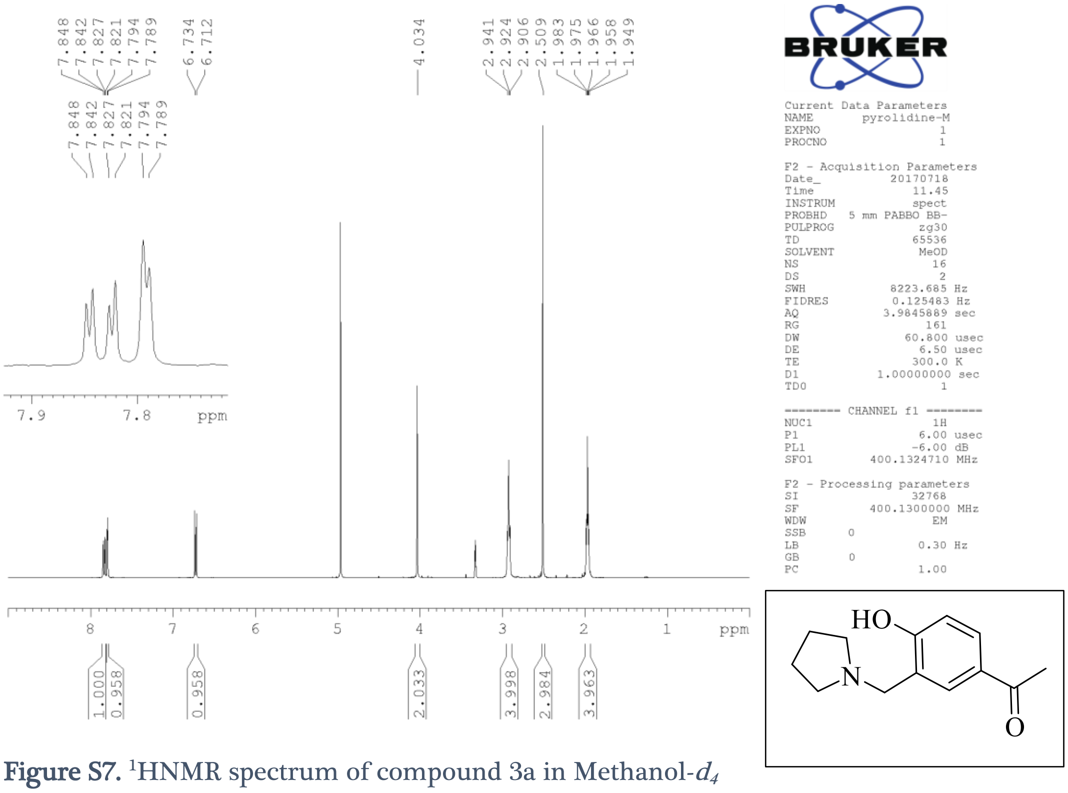

Figure S7. ^{1}HNMR spectrum of compound 3a in Methanol-d_4

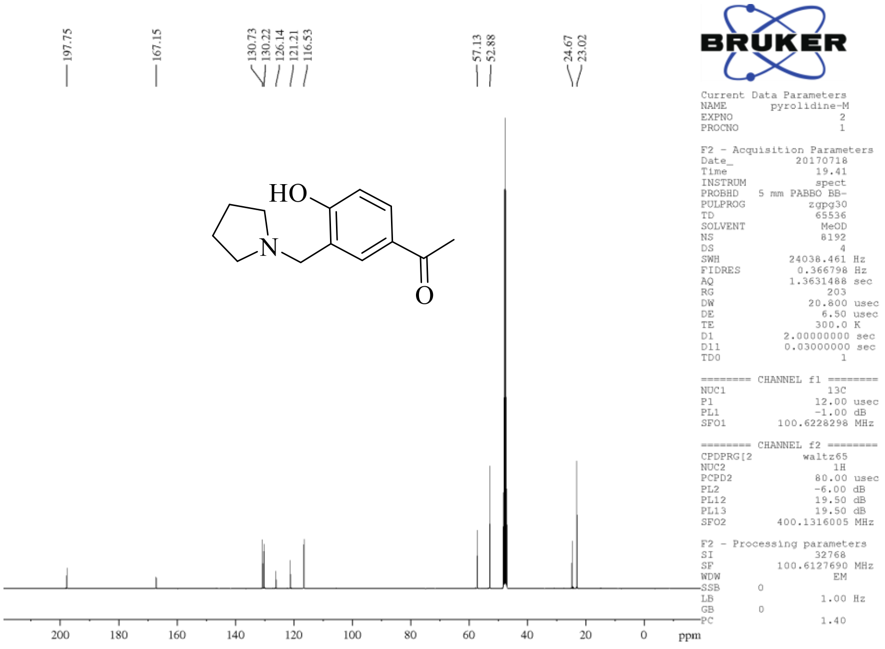

Figure S8. ^{13}C NMR spectrum of compound 3a in Methanol-d_4

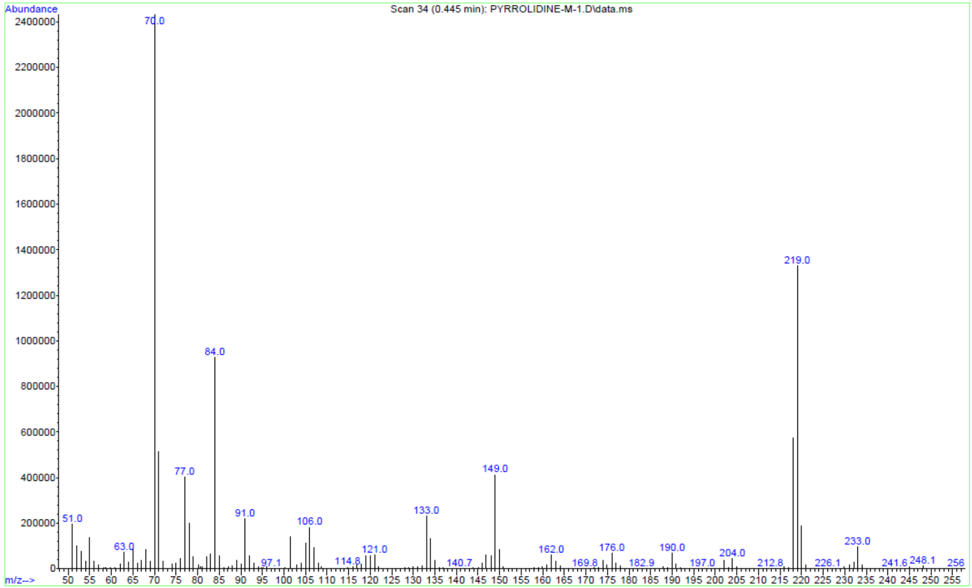

Figure S9. MS spectrum of compound 3a

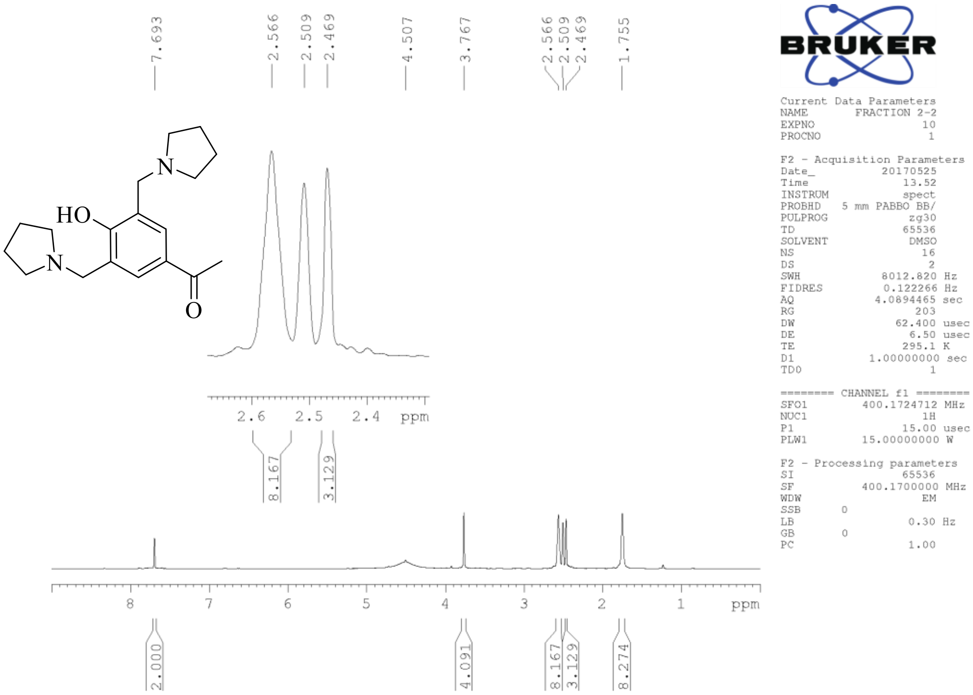

Figure S10. ^{1}HNMR spectrum of compound 3b in DMSO-d_6.

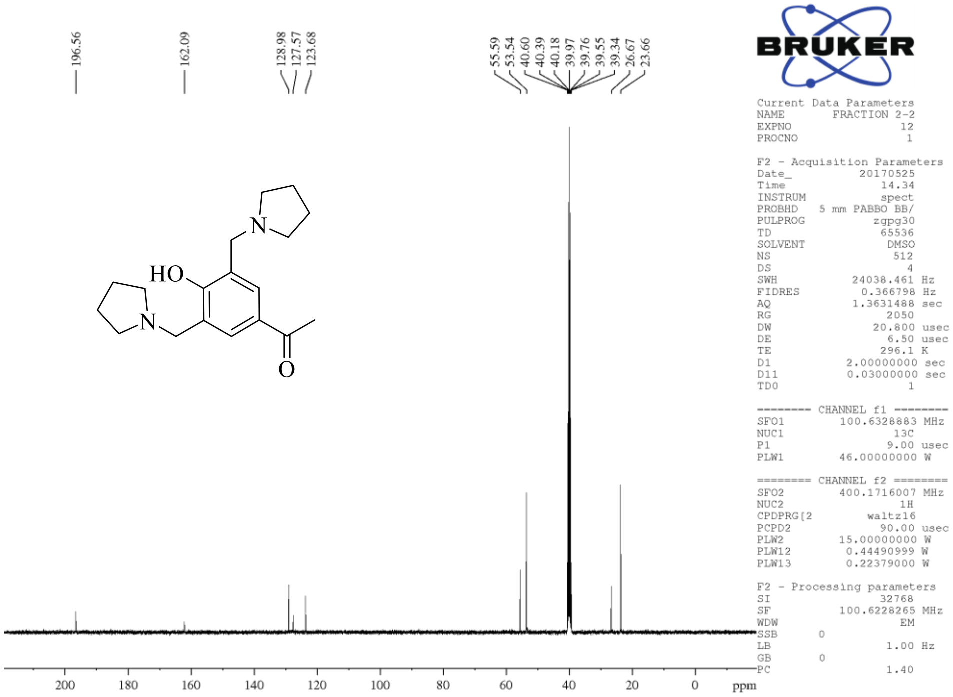

Figure S11. ^{13}C NMR spectrum of compound 3b in DMSO-d_6.

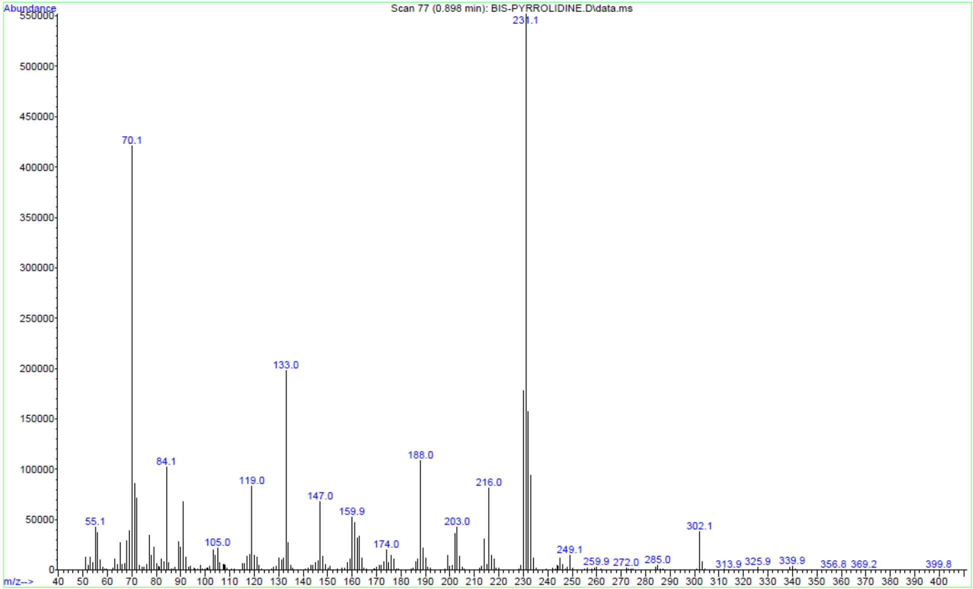

Figure S12. MS spectrum of compound 3b

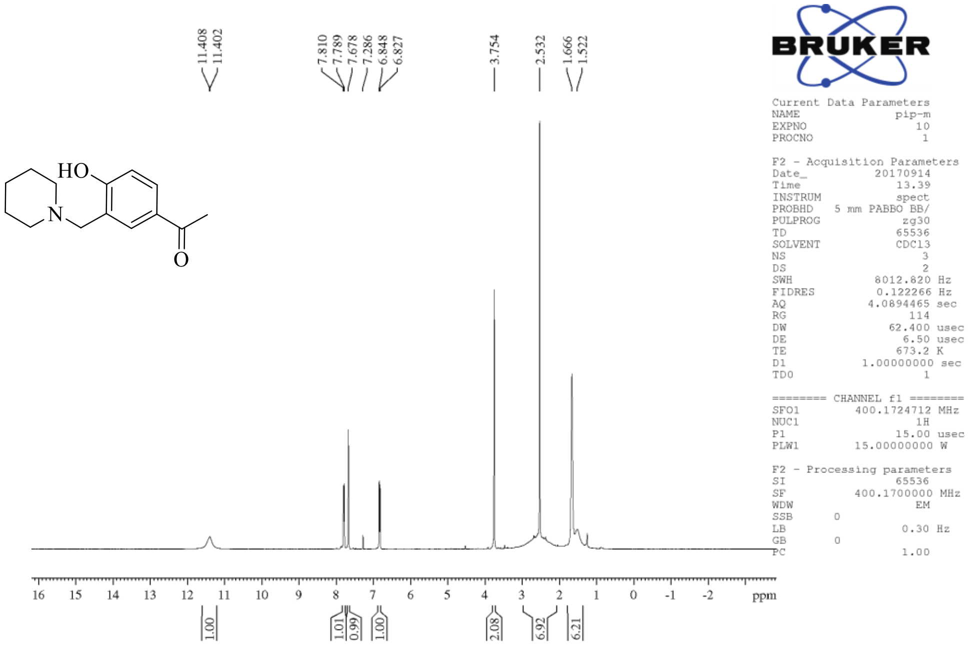

Figure S13. ^{1}HNMR spectrum compound 4a in $CDCl_3$.

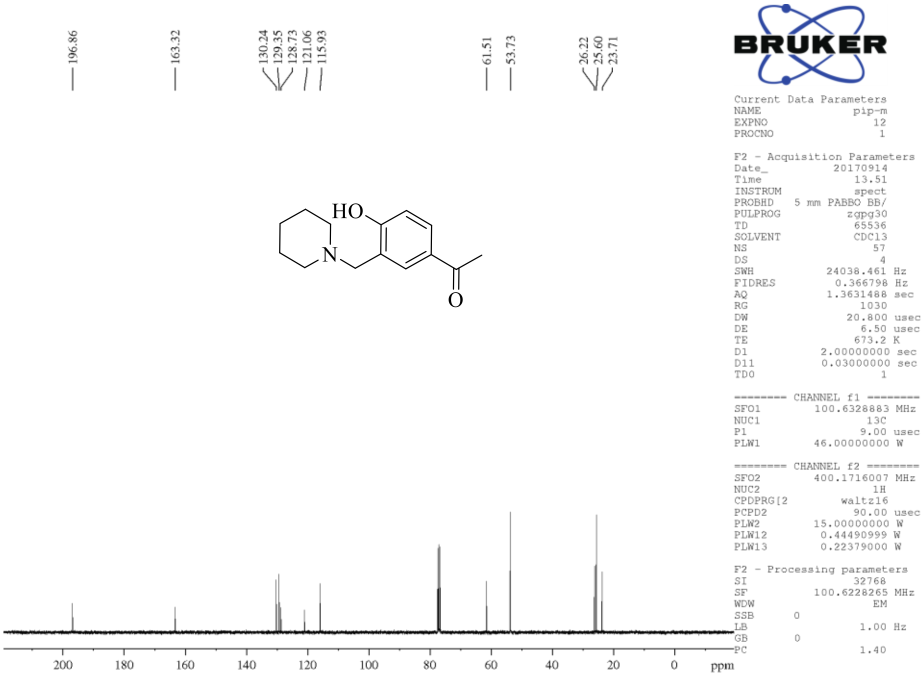

Figure S14. ^{13}C NMR spectrum compound 4a in $CDCl_3$.

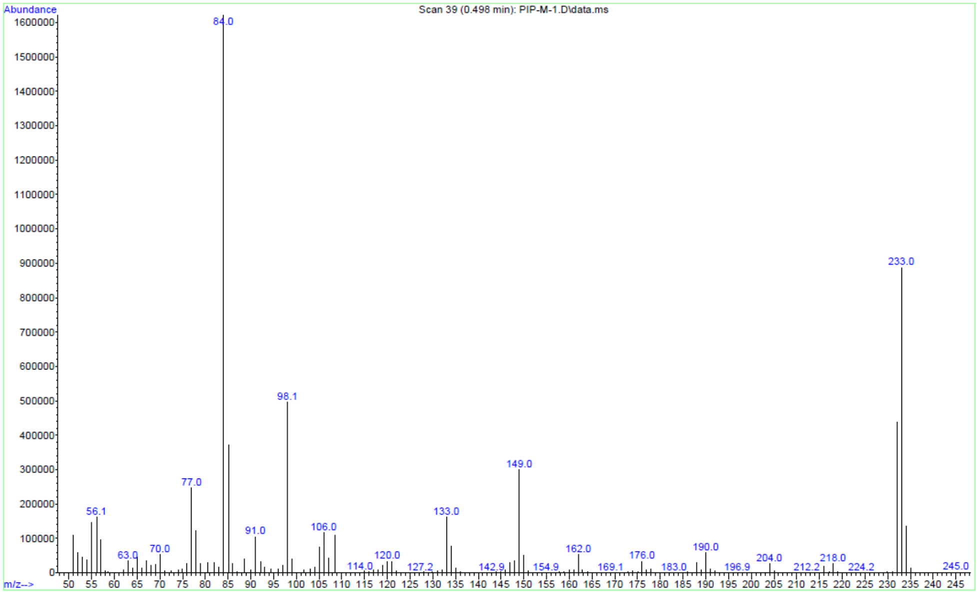

Figure S15. MS spectrum of compound 4a

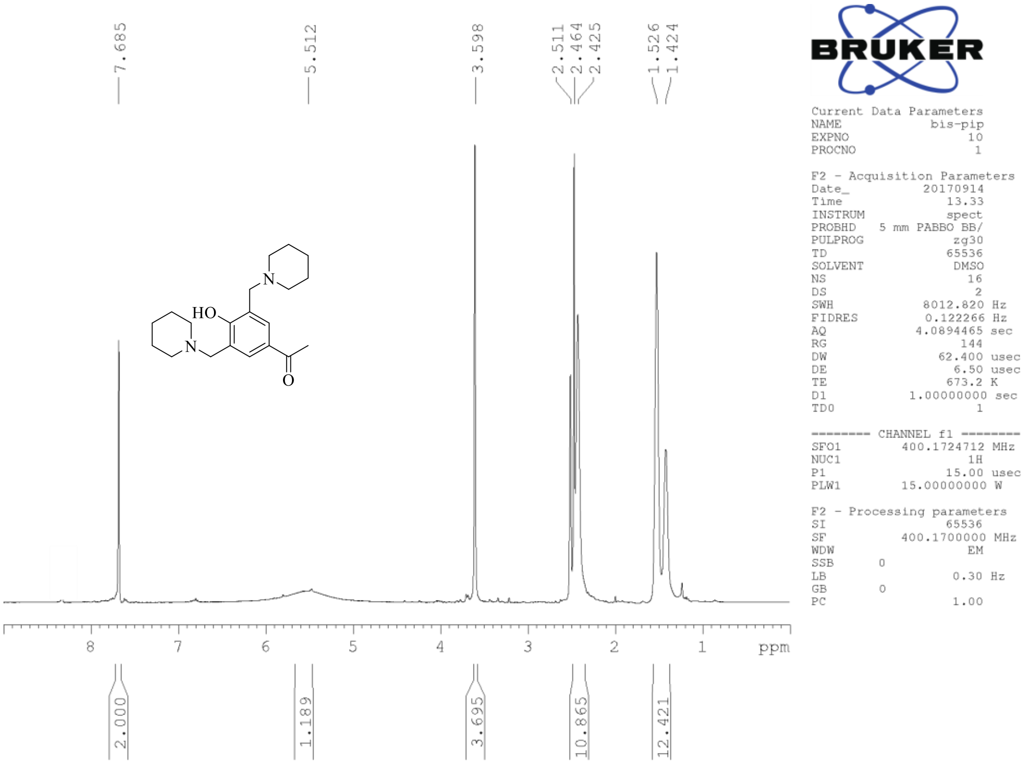

Figure S16. ^{1}HNMR spectrum of compound 4b in DMSO-d_6.

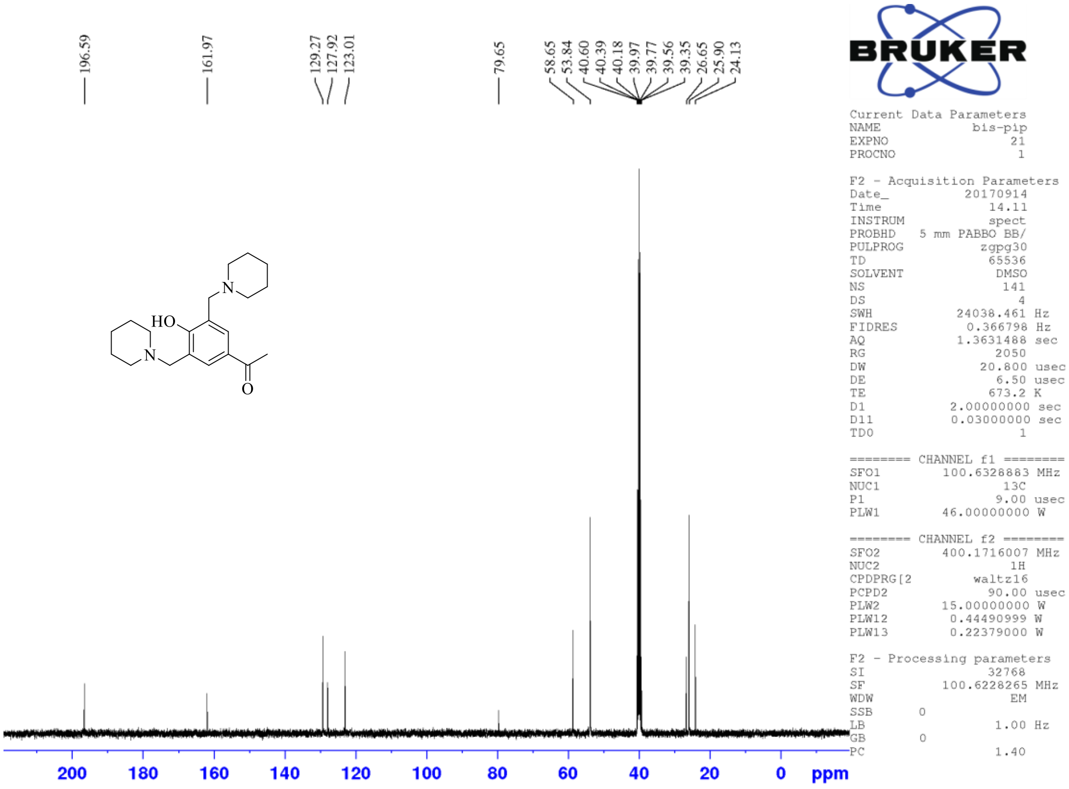

Figure S17. ^{13}C NMR spectrum of compound 4b in DMSO-d_6.

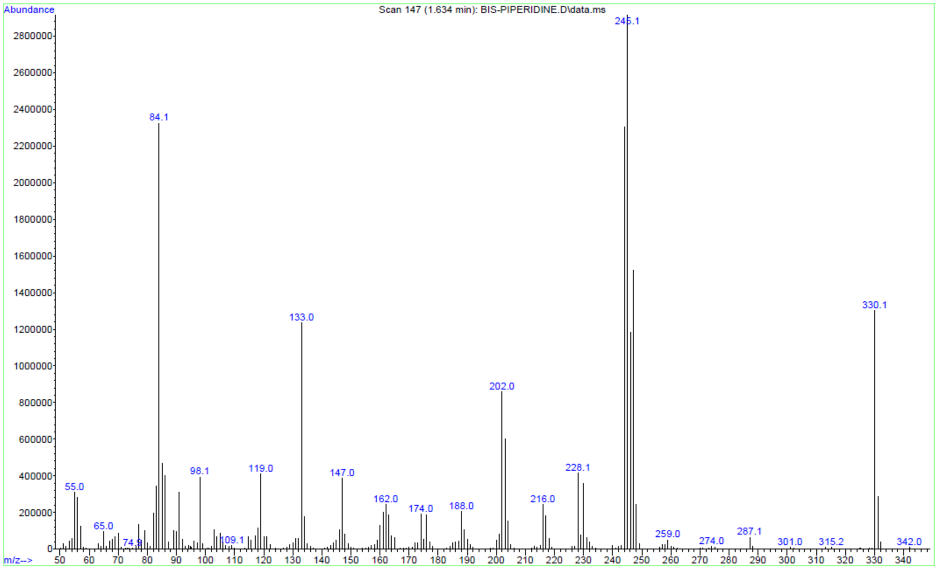

Figure S18. MS spectrum of compound 4b

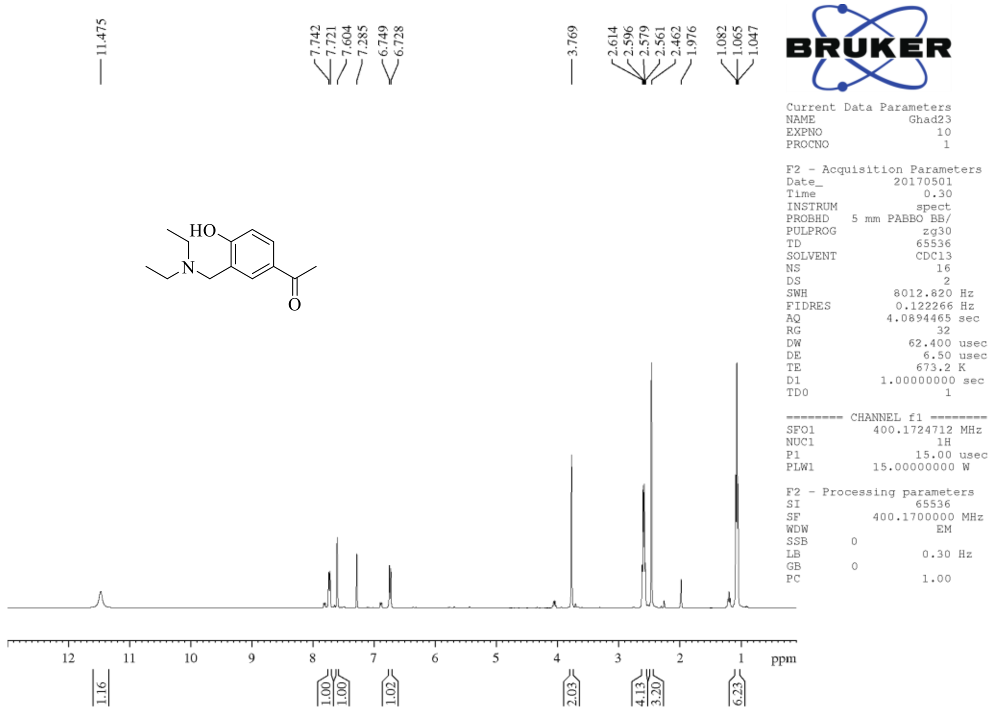

Figure S19. ^{1}HNMR spectrum of compound 5a in $CDCl_3$.

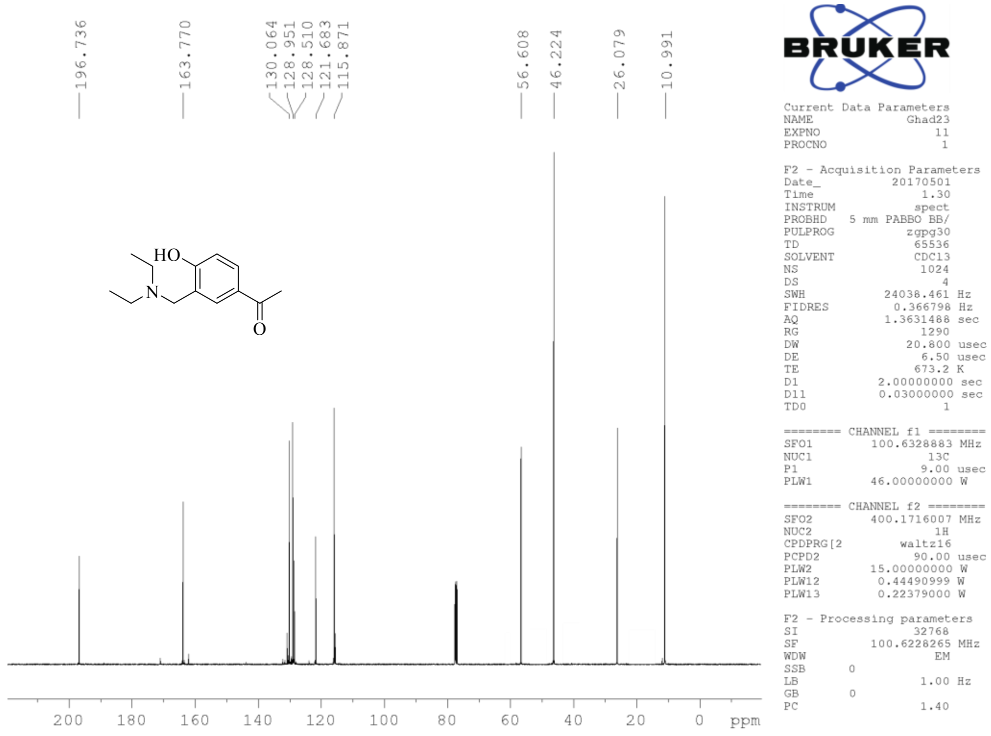

Figure S 20. ^{13}C NMR spectrum of compound 5a in $CDCl_3$.

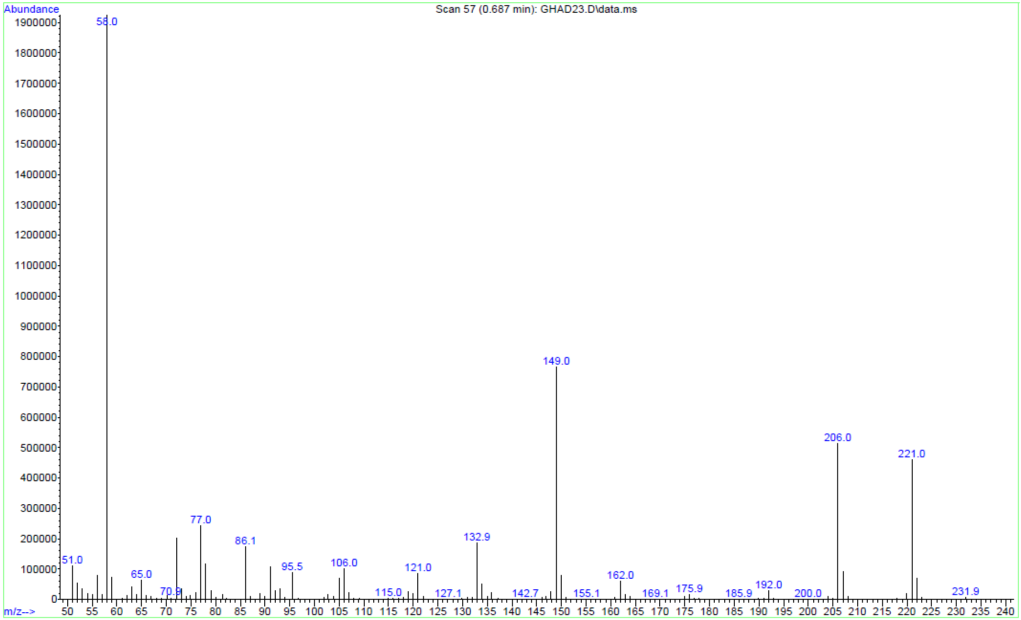

Figure S 21. MS spectrum of compound 5a
